# Comparison of Leaf Shape between a *Photinia* Hybrid and One of Its Parents

**DOI:** 10.3390/plants11182370

**Published:** 2022-09-11

**Authors:** Xiao Zheng, Karl J. Niklas, David A. Ratkowsky, Yabing Jiao, Hui Ding, Peijian Shi

**Affiliations:** 1 Research Center for Biodiversity Conservation and Biosafety, Nanjing Institute of Environmental Sciences, Ministry of Ecology and Environment of China, Nanjing 210042, China; 2 Bamboo Research Institute, College of Biology and the Environment, Nanjing Forestry University, Nanjing 210037, China; 3 School of Integrative Plant Science, Cornell University, Ithaca, NY 14853, USA; 4 Tasmanian Institute of Agriculture, University of Tasmania, Private Bag 98, Hobart 7001, Australia

**Keywords:** leaf ellipticalness index, leaf length, leaf width, Montgomery equation, Montgomery parameter

## Abstract

Leaf shape and size can vary between hybrids and their parents. However, this has seldom been quantitatively tested. *Photinia × fraseri* is an important landscaping plant in East Asia as a hybrid between evergreen shrubs *P. glabra* and *P. serratifolia*. Its leaf shape looks like that of *P. serratifolia*. To investigate leaf shape, we used a general equation for calculating the leaf area (*A*) of broad-leaved plants, which assumes a proportional relationship between *A* and product of lamina length (*L*) and width (*W*). The proportionality coefficient (which is referred to as the Montgomery parameter) serves as a quantitative indicator of leaf shape, because it reflects the proportion of leaf area *A* to the area of a rectangle with *L* and *W* as its side lengths. The ratio of *L* to *W*, and the ellipticalness index were also used to quantify the complexity of leaf shape for elliptical leaves. A total of >4000 leaves from *P. × fraseri* and *P. serratifolia* (with >2000 leaves for each taxon) collected on a monthly basis was used to examine: (i) whether there is a significant difference in leaf shape between the two taxa, and (ii) whether there is a monotonic or parabolic trend in leaf shape across leaf ages. There was a significant difference in leaf shape between the two taxa (*p* < 0.05). Although there were significant differences in leaf shape on a monthly basis, the variation in leaf shape over time was not large, i.e., leaf shape was relatively stable over time for both taxa. However, the leaf shape of the hybrid was significantly different from its parent *P. serratifolia*, which has wider and more elliptical leaves than the hybrid. This work demonstrates that variations in leaf shape resulting from hybridization can be rigorously quantified and compared among species and their hybrids. In addition, this work shows that leaf shape does not changes as a function of age either before or after the full expansion of the lamina.

## 1. Introduction

Leaves are the primary photosynthetic organs of the majority of land plants. Prior research has shown that leaf area and structure are correlated with photosynthetic rates [1,2,3,4]. It is important therefore to accurately calculate leaf area. Prior studies have shown that leaf area follows a general equation called the Montgomery equation (denoted henceforth as ME) for many broad-leaved species with different leaf shapes. This equation assumes a simple proportional relationship between leaf area (*A*) and the product of leaf length (*L*) and width (*W*) [5,6,7,8,9,10,11,12,13]. Leaf age and area have been demonstrated to have little influence on the validity of ME in calculating leaf area, although leaf age and size can influence to some degree the proportionality coefficient of ME among different leaf age or size groups [13,14].

Leaf shape and area are adaptively correlated and therefore can vary as a function of local climatic conditions even among conspecifics [15,16,17,18]. In previous studies, the leaf roundness index [19,20,21,22] has been widely used to measure leaf shape. However, this index is more suitable for leaves whose length and width are nearly equal. Consequently, Li et al. [23] proposed the ‘leaf ellipticalness index’ for elliptical and oblong leaves. For an elliptical leaf, the leaf ellipticalness index approximates 1. Additionally, in spite of the simplicity of using the leaf width/length ratio to quantify leaf shape, Shi et al. [24] have shown that the leaf width/length ratio is significantly positively correlated with the fractal dimension of the lamina perimeter based on a large sample of leaves differing in size and shape among nine Magnoliaceae species.

**Figure 1:**
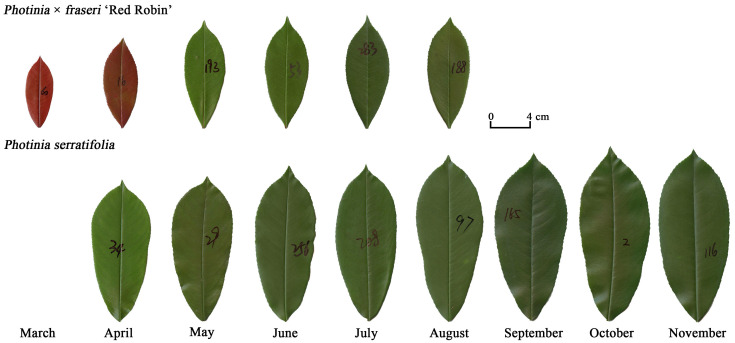
Representative leaves for the two *Photinia* taxa (the hybrid *P.* *× fraseri* and one of its parents *P. serratifolia*) at different times in the growing season during 2021.

Hybridization across closely related plant species can also result in significant variation in leaf shape. However, the effect of hybridization on leaf size and shape has seldom been quantitatively examined, particularly as leaves mature during the growing season. In order to examine this phenomenology, *Photinia × fraseri*, which is a hybrid between the evergreen shrubs *P. glabra* and *P. serratifolia*, and one of its parents *P. serratifolia*, were examined to determine (i) whether there is a significant difference in leaf shape between *P. × fraseri* and *P. serratifolia*, and (ii) whether there is a monotonic or parabolic trend in leaf shape as a function of age. Because both the hybrid and one of its parents have elliptical or obovate leaves (Figure 1), we used the ratio of leaf width to length and the leaf ellipticalness index to quantify leaf shape in addition to the ME.

## 2. Materials and Methods

### 2.1. Plant Materials

We marked newly emerging leaves on 36 trees of *P. × fraseri* (the hybrid) and 3 trees of *P. serratifolia* (one of the two parents) growing at the Nanjing Forestry University campus (118°48′35″ E, 32°4′67″ N) from late February to early March 2021. Representative specimens of the second parent (*P. glabra*) were not found for study on the University campus, which was under quarantine. Each month, 320–380 leaves were randomly sampled from three individual trees of *P. × fraseri* from mid-March to mid-August 2021 and 320–350 leaves were randomly sampled from three individual trees of *P. serratifolia* from mid-April to mid-November 2021. Figure 1 provides images of representative leaves across the different months for the two taxa. We had planned to sample the leaves of *P. serratifolia* from spring to autumn. However, the marked branches in early March were pruned by gardeners at the beginning of September 2021. Fortunately, the leaves growing from late February to early March 2021 were fully mature in August 2021, and the leaves sampled from mid-March to mid-August manifest temporal changes in leaf shape from young to mature leaves of this hybrid. When a leaf matures, its leaf shape seldom changes significantly.

### 2.2. Data Acquisition

The fresh leaves were scanned by a photo scanner (V550, Epson, Batam, Indonesia) to obtain .tiff images at a 600-dpi resolution, which were transferred to black-white .bmp files using the Photoshop software (CS6, version: 13.0; Adobe, San Jose, CA, USA). The Matlab (version ≥ 2009a; MathWorks, Natick, MA, USA) procedures developed by refs. [25,26] were used to extract the planar coordinates of each leaf boundary. The ‘biogeom’ package (version 1.1.1) [27] based on the statistical software R (version 4.2.0) [28] was then used to calculate leaf area, length, and width using the planar coordinates.

### 2.3. Methods

The Montgomery equation (ME) [5] was used to describe the relationship between leaf area (*A*) and the product of leaf length (*L*) and width (*W*):

(1) $$A=m_{p}\cdot LW,$$

where *m_p_* is the proportionality coefficient, which is the Montgomery parameter. To normalize the data, we log-transformed the data of both sides of Equation (1):

(2) y=a+x,

where *y* = ln(*A*); *x* = ln(*LW*); *a* = ln(*m_p_*). We used ordinary least-squares protocols to estimate the parameter *a*, and its 95% confidence interval (95% CI). To test whether there is a significant difference in *m_p_* between the two taxa, we used the bootstrap percentile method [29,30] to calculate the 95% CI of the differences in the 4000 bootstrapping replicates of *m_p_* between *P. × fraseri* and *P. serratifolia*. If the 95% CI of the differences includes 0, there is no significant difference in *m_p_* between the two taxa; if it does not include 0, a significant difference is detected. We used the Pearson’s product moment correlation coefficient (*r*) to measure the validity (i.e., statistical robustness) of the linear relationship between *x* and *y*:

(3) $$r=\frac{\sum_{i=1}^{n}\left(x_{i}-\bar{x}\right)\left(y_{i}-\bar{y}\right)}{\sqrt{\sum_{i=1}^{n}{\left(x_{i}-\bar{x}\right)}^{2}}\sqrt{\sum_{i=1}^{n}{\left(y_{i}-\bar{y}\right)}^{2}}},$$

where $\bar{x}$ and $\bar{y}$ represent the means of *x*- and *y*-values, respectively; and *n* is the sample size. The test of the significance of the correlation coefficient is based upon a statistic that theoretically follows a *t* distribution with *n* − 2 degrees of freedom if the samples of *x* and *y* are normally distributed. 

The leaf ellipticalness index (EI) was used to quantify leaf shape [23]:

(4) $$\mathrm{EI}=\frac{A}{\left(\pi /4\right)LW}.$$

The EI has a close relationship with *m_p_*, i.e., EI = *m_p_*/(π/4). A linear regression method was used to estimate the *m_p_* value based on the number of leaves. However, EI can also be calculated directly using *A*, *L* and *W*. The EI quantifies the extent of a leaf shape deviating from an ellipse regardless of the eccentricity of the ellipse. Thus, we used the *W/L* ratio as another leaf-shape index. ANOVA with Tukey’s honestly significant difference (HSD) test [31] and a 0.05 significance level were used to test the significance of the differences in EI as well as the *W/L* ratio between the two taxa at the combined data level and at the intraspecific level across different dates of collection. For any two groups, we calculated the 95% confidence interval (CI) of the Tukey’s test confidence interval, which equals the difference in the means between the two groups ± HSD, and we determined its significance by checking whether the 95% CI included 0. We also used the linear and parabolic equations to fit the EI (and the *W/L* ratio) vs. collection month data, where month was set to a numeric variable to test whether leaf shape has a monotonic or parabolic trend across different investigation dates (i.e., leaf ages).

(5) $$\mathrm{LS}=\beta_{0}+\beta_{1}\mathrm{Month}+\beta_{2}{\mathrm{Month}}^{2},$$

where LS is the leaf-shape index of interest (EI or the *W/L* ratio); Month is the month of sampling (ranging from 3 to 8 [i.e., March to August 2021] for *P. × fraseri*, and from 4 to 11 [i.e., April to November 2021] for *P. serratifolia*); and $\beta_{0}$, $\beta_{1}$ and $\beta_{2}$ are the parameters to be fitted (referred to as regression coefficients). For the simple linear regression, $\beta_{2}$ was set to be zero. Each regression coefficient’s statistic in Equation (5) follows a *t* distribution with *n* − *p* degrees of freedom, where *n* is the sample size and *p* is the number of parameters [32]. 

The statistical software R (version 4.2.0) [28] was used to carry out calculations and to draw figures. The ‘agricolae’ package (version 1.3-5) was used to implement the Tukey’s HSD test. 

## 3. Results

**Figure 2:**
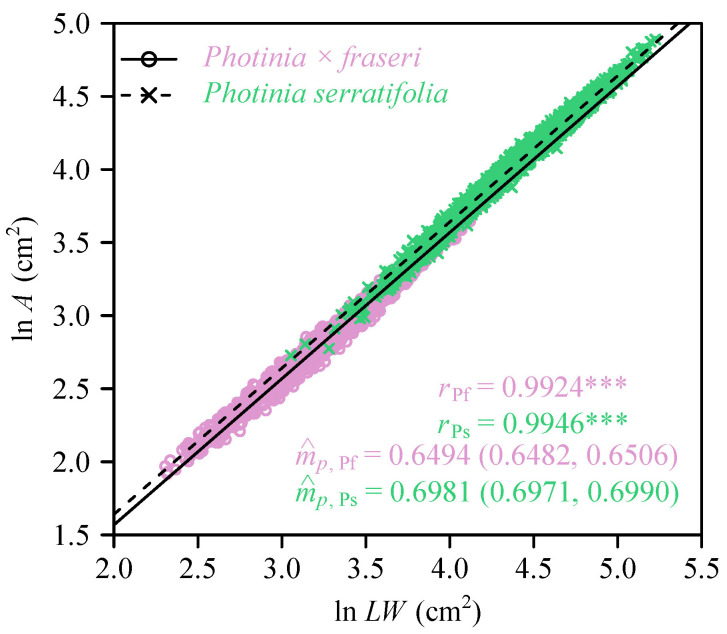
Log-log bivariate plot of leaf area (*A*) vs. the product leaf length (*L*) and width (*W*). The symbol ${\hat{m}}_{p}$ represents the estimated value of the Montgomery parameter, i.e., the estimated proportionality coefficient of the Montgomery equation. The subscripts Pf and Ps represent the hybrid *P.* *× fraseri*, and one of its parents *P. serratifolia*, respectively. *** denotes *p* < 0.001 for a correlation coefficient.

**Figure 3:**
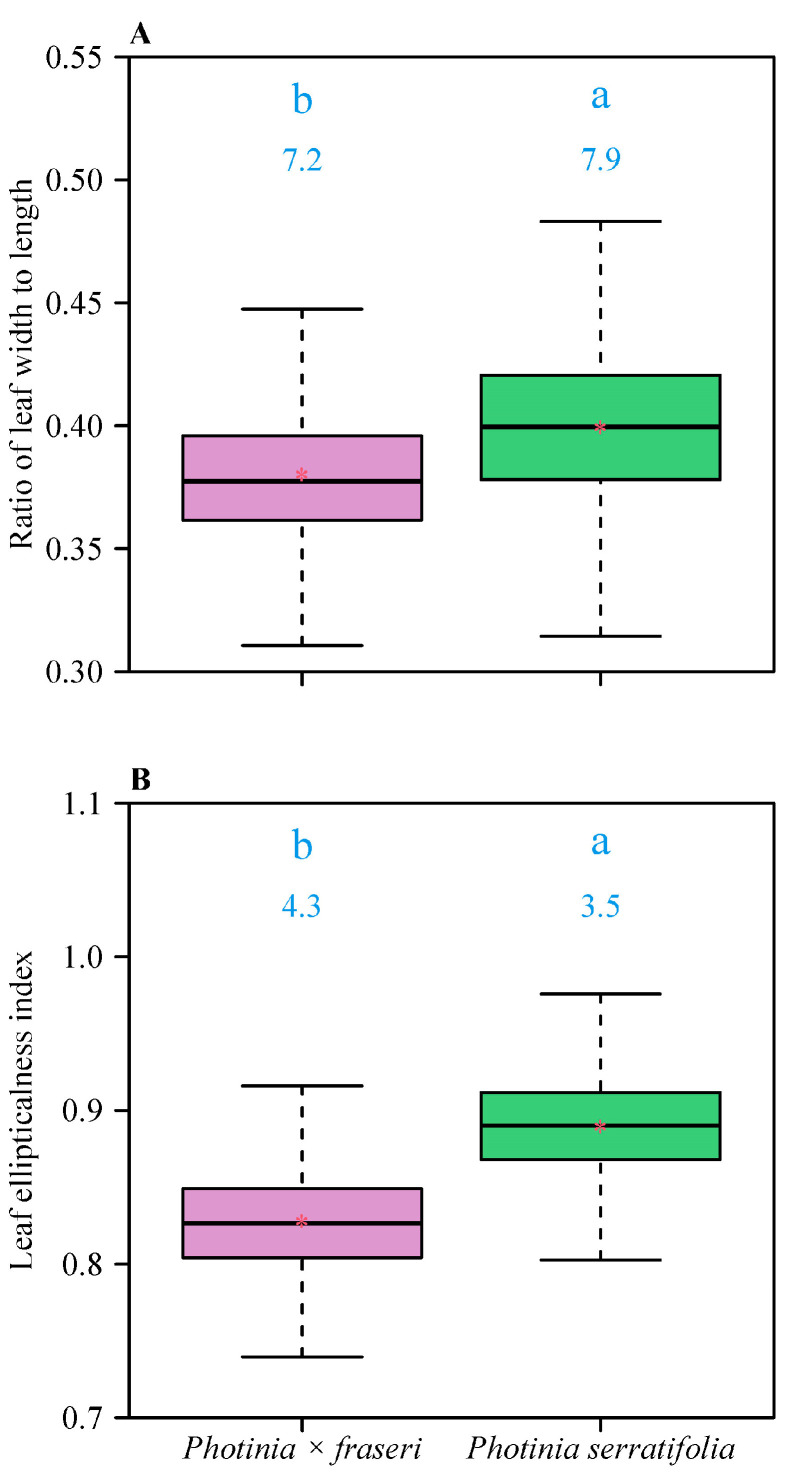
Comparison of leaf shape between the two *Photinia* taxa for the pooled data. (**A**) Ratio of leaf width to length; (**B**) Leaf ellipticalness index. In each panel, the letters on the whiskers show differences between the two taxa (i.e., taxa with different letters are significantly different at the α = 0.05 significance level using Tukey’s HSD); the values at the top of whiskers represent the coefficients of variation (%) for each taxon; the horizontal solid line represents the median; and the red asterisk represents the mean. The whiskers extend to the most extreme data point, which is no more than 1.5 times the interquartile range from the box. The difference in mean leaf *W/L* ratios is equal to 0.0193 with HSD = 0.0017, and the corresponding 95% Tukey’s test confidence interval is 0.0176, 0.0210, which does not include 0, indicating a significant difference; the difference in mean leaf ellipticalness indices is equal to 0.0617 with HSD = 0.0019, and the corresponding 95% Tukey’s test confidence interval is 0.0598, 0.0636, which also does not include 0, indicating a significant difference.

The Montgomery equation (ME) was valid in calculating leaf area. For the pooled intraspecific data, the correlation coefficient was greater than 0.99 (*p* < 0.001). The estimated values of the Montgomery parameters of *P. × fraseri* and *P. serratifolia* were 0.6494 (95% CI = 0.6482, 0.6506) and 0.6981 (95% CI = 0.6971, 0.6990), respectively (Figure 2). There was a significant difference in leaf shape between the two taxa, i.e., the leaves of *P. serratifolia* were on average wider and more elliptical in shape (Figure 3). The means of the *W/L* ratios for the two taxa were 0.38 (*P. × fraseri*) and 0.40 (*P. serratifolia*); the means of the leaf elliptical indices (EIs) for the two taxa were 0.8276 (*P. × fraseri*) and 0.8894 (*P. serratifolia*).

**Figure 4:**
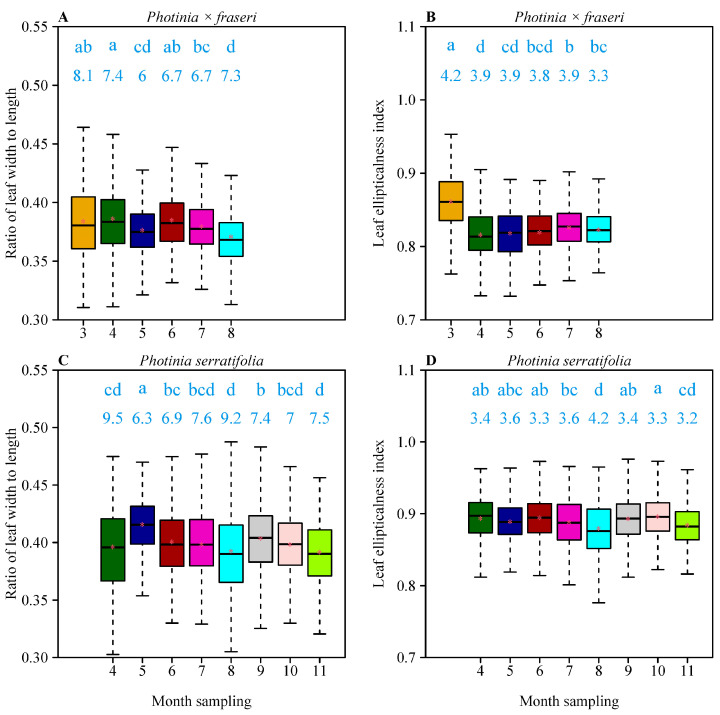
Comparison of leaf shapes (reflected by the ratio of leaf width to length and leaf ellipticalness index) across different sampling months for *P.* *× fraseri* (**A**,**B**) and *P. serratifolia* (**C**,**D**). In each panel, the letters on the whiskers represent the significance of the differences in leaf shape between any two sampling months among which the letters a, b, c, and d are used to represent the significance of differences in leaf shape among different sampling months; groups sharing a common letter are not significantly different at the 0.05 significance level; the values at the top of whiskers represent the coefficients of variation (%) for each sampling month; the horizontal solid line represents the median; the red asterisk represents the mean. The whiskers extend to the most extreme data point, which is no more than 1.5 times the interquartile range from the box. For the label of the *x*-axis, the months 3 to 11 represent March to November 2021, respectively. The differences in the means of leaf-shape indices, HSD values, and the corresponding 95% Tukey’s test confidence intervals between any two groups for *P.* *× fraseri* and *P. serratifolia* are provided in Tables S3 and S4, respectively.

There were significant differences in leaf shape (measured as either the *W/L* ratio or the EI) among the different months of collecting (Figure 4). Three of the four slopes of the linear model of leaf shape vs. month were statistically significant (with three *p* values < 0.05) with the exception of the EIs of *P. serratifolia*. However, the four coefficients of determination (i.e., *r*^2^ values) were all less than 0.06, i.e., a very weak linear relationship was observed between leaf shape and sampling month. Thus, there is no robust evidence for a monotonic increase or decrease in leaf shape across leaf ages. The linear and quadratic coefficients in the parabolic model were statistically significant (*p* values < 0.05) for the leaf *W/L* ratio vs. month relationship and the EI vs. month relationship for *P. × fraseri*. However, the two coefficients were not statistically significant (*p* values > 0.05) for any leaf-shape indices for *P. serratifolia*. Three of the four coefficients of determination of the parabolic regression were smaller than 0.03, with the exception of the EI vs. month data of *P. × fraseri* (*r*^2^ = 0.1452). Thus, there was also no strong evidence for a parabolic trend in leaf shape across the different leaf ages (see Table 1 for details).

## 4. Discussion and Conclusions

Shi et al. [33] used the Montgomery parameter (*m_p_*) to fit leaf *A* vs. *LW* of 101 bamboo taxa using > 10,000 bamboo leaves. In their study, the estimated value of *m_p_* was 0.6959 (95% CI = 0.6952, 0.6966). Li et al. [23] estimated a *m_p_* value of 0.6840 (95% CI = 0.6827, 0.9855) using > 2200 leaves from nine Magnoliaceae species, whereas Ma et al. [13] estimated a *m_p_* value of 0.7710 (95% CI = 0.7696, 0.7714) using > 6500 leaves of an alpine oak species (*Quercus pannosa*) from different tree size groups. The present study shows that the estimated value of *m_p_* of the two *Photinia* taxa also approximates 0.7, i.e., the leaf area approximately accounts for 70% of the area of a hypothetical rectangle with the leaf length and width as its two side lengths. This result is significantly different from the numerical value of the alpine oak provided in ref. [13], because the leaf shape of *Q. pannosa* is a special superellipse with a mean leaf ellipticalness index greater than unity [34,35,36], which is larger than those of the two *Photinia* taxa examined in this study. Since leaf shape is found to be closely related to climatic factors [18,37], it would be worth studying the link between the *m_p_* and climate for some widely distributed plants in the future.

The leaf shapes of two *Photinia* taxa were quantified using different metrics, and found to significantly differ in spite of the fact that the two types of leaves appear to be superficially very similar in appearance. The leaves of *P. serratifolia* are wider and more elliptical than those of its hybrid. In addition, leaf shape significantly varied across the different leaf ages, but manifested no monotonic or parabolic tendency across the different leaf ages. The variations in leaf shape among the different months of sampling may reflect variations resulting from random samplings of individual plants. This variation, however, does not mask the fact that the leaf shape of the *Photinia* taxa is relatively stable, and that the Montgomery equation, which assumes a proportional relationship between leaf area and the product of leaf length and width, is valid at different leaf growth stages. These results highlight an approach that permits the non-destructive estimation of leaf area, i.e., direct field measurements of leaf length and width are used to estimate lamina area by multiplying their product by a proportionality coefficient (*m_p_*).

## Figures and Tables

**Table 1:** Regression statistics of the leaf-shape index vs. sampling month.

Taxon/Leaf-Shape Index	Equation ^†^	Parameter	Estimate	Significance	*r* ^2^
*Photinia × fraseri* Leaf width/length ratio	*y* = *a* + *bx*	*a*	0.3920	<0.001	0.0192
		*b*	−0.0022	<0.001	
	*y* = *a* + *bx* + *cx*^2^	*a*	0.3733	<0.001	0.0233
		*b*	0.0054	0.0365	
		*c*	−0.0006	0.0031	
*Photinia × fraseri* Leaf ellipticalness index	*y* = *a* + *bx*	*a*	0.8549	<0.001	0.0591
		*b*	−0.0050	<0.001	
	*y* = *a* + *bx* + *cx*^2^	*a*	0.9668	<0.001	0.1452
		*b*	0.0504	<0.001	
		*c*	0.0041	<0.001	
*Photinia serratifolia* Leaf width/length ratio	*y* = *a* + *bx*	*a*	0.4096	<0.001	0.0099
		*b*	−0.0014	<0.001	
	*y* = *a* + *bx* + *cx*^2^	*a*	0.3974	<0.001	0.0111
		*b*	0.0022	0.268	
		*c*	−0.0002	0.071	
*Photinia serratifolia* Leaf ellipticalness index	*y* = *a* + *bx*	*a*	0.8932	<0.001	0.0014
		*b*	−0.0005	0.0562	
	*y* = *a* + *bx* + *cx*^2^	*a*	0.9010	<0.001	0.0018
		*b*	−0.0028	0.166	
		*c*	0.0002	0.252	

^†^ Here, *y* represents a specific leaf-shape index, and *x* represents the sampling month.

## Data Availability

The data used in the present work have been listed in the online Supplementary Materials.

## Supplementary Materials

The following supporting information can be downloaded at: https://www.mdpi.com/article/10.3390/plants11182370/s1, Table S1: The raw leaf data of *Photinia* *× fraseri*; Table S2: The raw leaf data of *P. serratifolia*; Table S3: Tukey multiple comparisons of the means of leaf-shape indices between any sampling months for *P. × fraseri*; Table S4: Tukey multiple comparisons of the means of leaf-shape indices between any sampling months for *P. serratifolia*.

## References

[1] Bhagsari A.S., Brown R.H. (1986). Leaf photosynthesis and its correlation with leaf area. Crop Sci..

[2] Reich P.B., Ellsworth D.S., Walters M.B. (2002). Leaf structure (specific leaf area) modulates photosynthesis–nitrogen relations: Evidence from within and across species and functional groups. Funct. Ecol..

[3] Wu Y., Gong W., Wang Y., Yong T., Yang F., Liu W., Wu X., Du J., Shu K., Liu J. (2018). Leaf area and photosynthesis of newly emerged trifoliolate leaves are regulated by mature leaves in soybean. J. Plant Res..

[4] Shi P., Miao Q., Niinemets Ü., Liu M., Li Y., Yu K., Niklas K.J. (2022). Scaling relationships of leaf vein and areole traits versus leaf size for nine Magnoliaceae species differing in venation density. Am. J. Bot..

[5] Montgomery E.G. (1911). Correlation Studies in Corn, Annual Report No.24.

[6] Kemp C.D. (1960). Methods of estimating leaf area of grasses from linear measurements. Ann. Bot..

[7] Stickler F.C., Wearden S., Pauli A.W. (1961). Leaf area determination in grain sorghum. Agronony.

[8] Jani T.C., Misra D.K. (1966). Leaf area estimation by linear measurements in *Ricinus communis*. Nat..

[9] Palaniswamy K.M., Gomez K.A. (1974). Length-width method for estimating leaf area of rice. Agron. J..

[10] Shi P., Liu M., Ratkowsky D.A., Gielis J., Su J., Yu X., Wang P., Zhang L., Lin Z., Schrader J. (2019). Leaf area-length allometry and its implications in leaf-shape evolution. Trees Struct. Funct..

[11] Yu X., Shi P., Schrader J., Niklas K.J. (2020). Nondestructive estimation of leaf area for 15 species of vines with different leaf shapes. Am. J. Bot..

[12] Schrader J., Shi P., Royer D.L., Peppe D.J., Gallagher R.V., Li Y., Wang R., Wright I.J. (2021). Leaf size estimation based on leaf length, width and shape. Ann. Bot..

[13] Ma J., Niklas K.J., Liu L., Fang Z., Li Y., Shi P. (2022). Tree size influences leaf shape but does not affect the proportional relationship between leaf area and the product of length and width. Front. Plant Sci..

[14] Huang L., Niinemets Ü., Ma J., Schrader J., Wang R., Shi P. (2021). Plant age has a minor effect on non-destructive leaf area calculations in moso bamboo (*Phyllostachys edulis*). Symmetry.

[15] Webb L.J. (1968). Environmental relationships of the structural types of Australian rain forest vegetation. Ecology.

[16] Lewis M.C. (1972). The physiological significance of variation in leaf structure. Sci. Prog..

[17] Givnish T., Solbrig O.T., Jain S., Johnson G.B., Raven P.H. (1979). On the adaptive significance of leaf form. Topics in Plant Population Biology.

[18] Royer D.L., Wilf P. (2006). Why do toothed leaves correlate with cold climates? Gas exchange at leaf margins provides new insights into a classic paleotemperature proxy. Int. J. Plant Sci..

[19] Baxes G.A. (1994). Digital Image Processing: Principles and Applications.

[20] Niinemets Ü., Cescatti A., Christian R. (2004). Constraints on light interception efficiency due to shoot architecture in broad-leaved Nothofagus species. Tree Physiol..

[21] Niinemets Ü., Sparrow A., Cescatti A. (2005). Light capture efficiency decreases with increasing tree age and size in the southern hemisphere gymnosperm Agathis australis. Trees Struct. Funct..

[22] Roth-Nebelsick A., Konrad W. (2019). Fossil leaf traits as archives for the past—And lessons for the future?. Flora.

[23] Li Y., Quinn B.K., Niinemets Ü., Schrader J., Gielis J., Liu M., Shi P. (2022). Ellipticalness index—A simple measure of the complexity of oval leaf shape. Pak. J. Bot..

[24] Shi P., Yu K., Niinemets Ü., Gielis J. (2021). Can leaf shape be represented by the ratio of leaf width to length? Evidence from nine species of *Magnolia* and *Michelia* (Magnoliaceae). Forests.

[25] Shi P., Ratkowsky D.A., Li Y., Zhang L., Lin S., Gielis J. (2018). A general leaf-area geometric formula exists for plants—Evidence from the simplified Gielis equation. Forests.

[26] Su J., Niklas K.J., Huang W., Yu X., Yang Y., Shi P. (2019). Lamina shape does not correlate with lamina surface area: An analysis based on the simplified Gielis equation. Glob. Ecol. Conserv..

[27] Shi P., Gielis J., Quinn B.K., Niklas K.J., Ratkowsky D.A., Schrader J., Ruan H., Wang L., Niinemets Ü. (2022). ‘biogeom’: An R package for simulating and fitting natural shapes. Ann. N. Y. Acad. Sci..

[28] R Core Team (2022). R: A Language and Environment for Statistical Computing.

[29] Efron B., Tibshirani R.J. (1993). An Introduction to the Bootstrap.

[30] Sandhu H.S., Shi P., Kuang X., Xue F., Ge F. (2011). Applications of the bootstrap to insect physiology. Fla. Entomol..

[31] Hsu J.C. (1996). Multiple Comparisons: Theory and Methods.

[32] Xue Y., Chen L. (2007). Statistical Models and R Software.

[33] Shi P., Li Y., Niinemets Ü., Olson E., Schrader J. (2021). Influence of leaf shape on the scaling of leaf surface area and length in bamboo plants. Trees Struct. Funct..

[34] Lamé G. (1818). Examen des Différentes Méthodes Employées Pour Résoudre les Problèmes de Géométrie.

[35] Shi P., Huang J., Hui C., Grissino-Mayer H.D., Tardif J.C., Zhai L., Wang F., Li B. (2015). Capturing spiral radial growth of conifers using the superellipse to model tree-ring geometric shape. Front. Plant Sci..

[36] Huang W., Li Y., Niklas K.J., Gielis J., Ding Y., Cao L., Shi P. (2020). A superellipse with deformation and its application in describing the cross-sectional shapes of a square bamboo. Symmetry.

[37] Peppe D.J., Royer D.L., Gariglino B., Oliver S.Y., Newman S., Leight E., Enikolopov G., Fernandez-Burgos M., Herrera F., Adams J.M. (2011). Sensitivity of leaf size and shape to climate: Global patterns and paleoclimatic applications. New Phytol..

